# One‐Dimensional Earth‐Abundant Nanomaterials for Water‐Splitting Electrocatalysts

**DOI:** 10.1002/advs.201600380

**Published:** 2016-12-27

**Authors:** Jun Li, Gengfeng Zheng

**Affiliations:** ^1^ Laboratory of Advanced MaterialsDepartment of ChemistryCollaborative Innovation Center of Chemistry for Energy MaterialsFudan UniversityShanghai200433China

**Keywords:** bifunctional catalysts, electrocatalysts, hydrogen evolution reaction, oxygen evolution reaction, water splitting

## Abstract

Hydrogen fuel acquisition based on electrochemical or photoelectrochemical water splitting represents one of the most promising means for the fast increase of global energy need, capable of offering a clean and sustainable energy resource with zero carbon footprints in the environment. The key to the success of this goal is the realization of robust earth‐abundant materials and cost‐effective reaction processes that can catalyze both hydrogen evolution reaction (HER) and oxygen evolution reaction (OER), with high efficiency and stability. In the past decade, one‐dimensional (1D) nanomaterials and nanostructures have been substantially investigated for their potential in serving as these electrocatalysts for reducing overpotentials and increasing catalytic activity, due to their high electrochemically active surface area, fast charge transport, efficient mass transport of reactant species, and effective release of gas produced. In this review, we summarize the recent progress in developing new 1D nanomaterials as catalysts for HER, OER, as well as bifunctional electrocatalysts for both half reactions. Different categories of earth‐abundant materials including metal‐based and metal‐free catalysts are introduced, with their representative results presented. The challenges and perspectives in this field are also discussed.

## 1 Introduction

With the fast consumption of fossil fuels and gradual deterioration of global climate and environment situations due to carbon release, the research and development of clean and sustainable energy resources such as solar and wind energies have the promise of satisfying the energy need with minimum carbon footprint in the environment.^1^, ^2^, ^3^, ^4^, ^5^ Hydrogen has been well recognized as one of the most abundant and promising candidates for clean energy supply to replace fossil fuels since the beginning of 1970's, as the only combustion product is water.^6^, ^7^, ^8^, ^9^, ^10^, ^11^, ^12^, ^13^ Although hydrogen is one of the most abundant elements in the world, free hydrogen molecules do not exist naturally. Different synthetic methods have been investigated to obtain hydrogen fuel, among which the most predominant approach used in industry today is to passing steam through hydrocarbons to produce H_2_ and CO_2_.^6^ The electrical water splitting represents ≈ 4% contribution of global hydrogen fuel production to date. Compared to other approaches, as the chemical reaction of water splitting is indeed the reverse reaction of hydrogen combustion, this process enables the complete and close cycle of hydrogen with zero carbon emission, and is thereby regarded as a substantially important and promising method.^8^, ^9^, ^10^, ^11^, ^12^, ^13^, ^14^

Firstly observed in 1789, the electrochemical water splitting is typically processed in an electrolyzer, which contains a cathode and an anode for the water reduction and oxidation half reactions, respectively.^1^, ^5^ An electrolyte is also used for transporting charges or ions between the cathode and anode. The overall chemical reaction is summarized as: H_2_O → H_2_ + 1/2 O_2_, although the two half reactions can be presented differently, depending on whether the reactions take place in acidic or neutral/basic electrolytes.^15^ The water splitting reaction is an energy uphill process, and the standard‐state free energy change (Δ*G* ^o^) for converting one mole of water molecules into hydrogen and oxygen gases is +237.2 kJ mol^–1^. When also taking the extra work for gas formation into consideration, the enthalpy change (Δ*H* ^o^) required is +286 kJ mol^–1^ that accounts for 1 mol of H_2_ formation. The thermodynamic electrical potential for a reversible electrolysis cell voltage is 1.23 V.^6^ Theoretically, when the applied potential across cathode and anode exceeds 1.23 V, the water reduction and oxidation take place, leading to the formation of hydrogen and oxygen gases that are released from the electrode surface. Nonetheless, in reality, an extra of voltage, known as the overpotential (η), is always needed to apply for water splitting to proceed with reasonable reaction rates. An electrochemical reaction is often assessed by determining current flowing through an electrode as a function of applied potential (i.e., by recording *i‐E* curves).^16^ The onset of the electrode (or cell) potential from the equilibrium value upon passage of faradaic current is known as polarization, which is regarded as the *overpotential, η*, a key parameter to evaluate electrocatalytic activity.

(1) $$\eta =E-E_{eq}$$

Typically in the order of several hundred of millivolts, this overpotential enables the electron transfer process to overcome the high activation energy of kinetic barriers, which are accounted for the formation of reaction intermediates on the electrode surface.^17^

**Figure 1:**
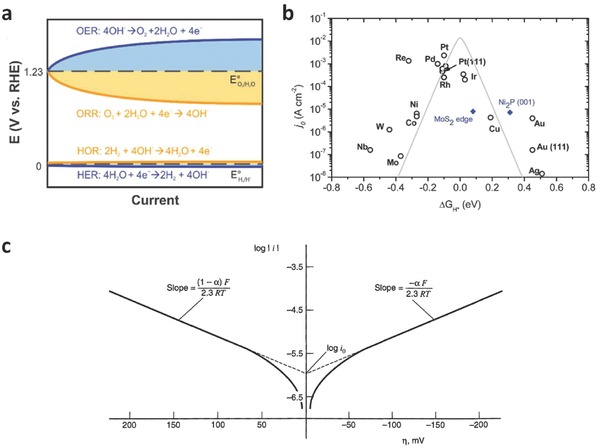
a) Schematic of the overpotentials associated with oxygen electrocatalysis (OER, ORR) and hydrogen electrocatalysis (HER, HOR). Reproduced with permission.^18^ Copyright 2015, Royal Society of Chemistry. b) A volcano plot of experimentally measured exchange current density as a function of the DFT‐calculated Gibbs free energy of adsorbed atomic hydrogen. Reproduced with permission.^6^ Copyright 2014, Royal Society of Chemistry. c) Tafel plots for anodic and cathodic branches of the current‐overpotential curve, with α = 0.5, T = 298 K, and j_0_ = 10^–6^ A cm^–2^. Reproduced with permission.^[16]^

The overpotentials associated with the water reduction half‐reaction (also known as hydrogen evolution reaction, HER) and the water oxidation half‐reaction (also known as oxygen evolution reaction, OER) are plotted with corresponding current densities (**Figure** **1**a, blue curves).^18^ Their reversed reactions, that is, the oxygen reduction reaction (ORR) and hydrogen oxidation reaction (HOR) are also displayed in this plot for comparison (Figure 1a, orange curves).^18^ The existence of overpotentials inevitably results in the cost of extra energy and lower conversion efficiency, and thereby catalysts are often used to incorporate on the electrode surface to reduce overpotential and increase the charge transfer towards water molecules. Generally, the electrocatalysts provide four main functions for water splitting: (i) stabilizing charge carriers (electrons and holes) and preventing them from recombination; (ii) offering adsorption sites for hydrogen and oxygen molecules; (iii) lowering the activation energies for water oxidation and reduction; and (iv) inhibiting the corrosion of the underlying semiconductor photoabsorbers. Thus, the major challenge in realizing hydrogen fuel for sustainable and clean energy conversion is the development of robust and scalable electrocatalysts, which allow for driving the electrochemical water splitting reactions with a high efficiency over a reasonably long period of time.

For the water reduction half‐reaction (HER) in cathode, platinum (Pt) has still been widely used and regarded as the benchmark for the HER catalytic activity.^13^ For the water oxidation half‐reaction (OER) in anode, ruthenium (Ru) or iridium (Ir) based oxides have been mostly used with the best OER activities.^8^ Nonetheless, as these noble metal‐based materials are scarce and associated with high cost and limited sustainability, the large scale deployment of these catalysts are not feasible. In addition, compared to HER that is relatively kinetically favorable, the OER is much more sluggish and requires much higher overpotentials to drive the electrochemical reaction, which limits the overall water splitting efficiency and its wide applications. A similar challenge also exists for ORR^19^, ^20^ and its related applications like in fuel cells and metal‐air batteries.^21^, ^22^ Moreover, most of the water splitting electrolyzers today are functioned in alkaline solutions, due to the general instability of metal oxides as OER catalysts in acidic conditions. Nonetheless, most of the HER catalysts (e.g. Pt and MoS_2_) have much better performances in acidic electrolytes. This mismatched pH condition inevitably reduces the device efficiencies and increases the fabrication cost.^23^

The research and development of earth‐abundant materials for HER and OER electrocatalysts have attracted substantial interest in the past decade, with a variety of catalyst candidates being explored and developed. For the HER catalysts, transition metal sulfides, selenides, carbide, nitride, phosphide, and heteroatom‐doped carbon have been reported.^15^ For the OER catalysts, the effort of using earth‐abundant catalysts has been mainly focused on three categories of materials:^18^ Co, Ni, or Mn‐based oxides, mixed oxides with spinel or perovskite structures, and Co, Ni‐containing molecular complex or macrocycles. In general, an ideal electrocatalyst should have the following features: (1) high efficiency that is similar to noble metal (oxide or hydroxide) based materials; (2) high chemical and catalytic activity over a wide range of pHs; (3) good durability with stable activity for a long time (months even years); (4) abundant sources and low cost; (5) environmental friendly; (6) scalability for commercial deployment; (7) potentials of integration for both HER and OER catalysts as well as integration with photoabsorbers like semiconductors.

The research of new electrocatalysts for water splitting has also been accompanied by the fast development of theoretical understanding of catalytic functions on the surface. Different parameters of materials in performing catalytic functions have been investigated, and summarized with their performances such as exchange current densities (*j_0_*). For instance, it has been proposed that the intrinsic activity of an HER catalyst is well correlated with the Gibbs free energy for hydrogen adsorption (Δ*G*_H*_) on a catalyst surface.^24^ For OER, one universal parameter is the difference between the adsorption free energies change between reaction intermediates of O* and OH* (i.e., ΔE_O*_ – ΔE_OH*_).^18^ By plotting the exchange current density of different materials with those structural parameters such as Δ*G*_H*_, a triangular shape can be observed and is known as the volcano plot (Figure 1b), which indicates that both the weak and strong interactions between the reactant species and the catalyst surface are not favorable for the optimal catalytic activity. Thus, for an ideal catalyst, the system needs to attain to the top of the activity volcano plot, which should be observed experimentally with the highest value of the exchange current density. For example, it can be seen that Pt group metals are in the top of the HER volcano.

**Figure 2:**
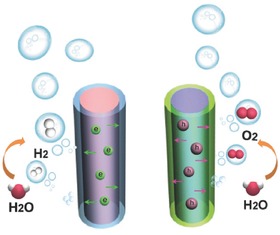
Scheme of benefits for one‐dimensional nanoscale materials as one unique target for serving excellent electrocatalyst candidates.

In addition to the catalyst material selection, the design and fabrication of nanostructured electrocatalysts have been driving a significant increase of efficiency in the past decade, either by preferentially exposing high‐index facets or active reaction sites, and/or electrically connecting these sites for fast charge transport/transfer. Thus, the morphology and structure of the designed catalysts can also provide significant benefits for their performances.^6^, ^25^ Among different types of morphologies and structures, the one‐dimensional (1D) nanoscale materials have been demonstrated as one unique target for serving excellent electrocatalyst candidates (**Figure** **2**).^26^, ^27^, ^28^ First, these 1D materials have high surface areas, large roughness factors and high active‐site densities, which are beneficial for providing efficient catalytic activity for surface electrochemical reactions. Second, the 1D materials can provide channels and few crystal boundaries for fast charge transport pathways with reduced scattering. Third, the existence of abundant open space and porosity between adjacent 1D nanostructures enables fast mass transport and chemical accessibility of electrolyte molecules into the deep portion of the electrode/catalyst surface. Fourth, the 1D materials can be directly grown and contacted on the underlying electrode surface, and thus provide efficient charge transport with low contact resistance, as well as waive the need of adding conducting additives or binders. Fifth, the formation and release of bubbles can also be facilitated from the 1D nanomaterial electrode/catalyst surface, thus preventing the bubbles blocking of subsequent reaction process.

The 1D nanomaterials have been discovered and become prosperous in the past two decades. Substantially research efforts have been invested into the synthesis of these 1D materials, which allow for exquisite controls of composition, morphology, heterostructures, and reactivity.^28^, ^29^ The capability of modulation the composition and doping, either radially or even longitudinally, even within a single 1D nanostructure has been explicitly demonstrated.^30^, ^31^ Thus, these achievements have also brought substantial advances and breakthroughs in the applications of utilization of 1D materials in almost all fields of modern technologies,^29^, ^32^, ^33^, ^34^ including the water splitting electrocatalysts.

In the following sections, we will first describe the representative progresses in studying 1D materials for HER and OER catalysts, respectively, with their representative examples including metal‐based and metal‐free materials illustrated. Then we will discuss the recent development of bifunctional HER/OER electrocatalysts for both water reduction and oxidation simultaneously. Finally, we will summarize these studies of electrocatalyst materials and structures, and suggest the challenges and future perspectives in this field.

## 2 HER

The mechanism of the HER process has been extensively studied in past decades, which can be divided into two steps.^35^ The first step is known as Volmer reaction, in which a proton adsorption on an empty active site of the electrode is coupled with an electron transfer, and yields an adsorbed hydrogen (H_ads_). In acidic or alkaline solutions, the proton source is the hydronium ion or a water molecule, respectively.^15^

(2) $$H_{3}O^{+}+e^{-}+M⇄H_{\mathrm{ads}}+H_{2}O,\mathrm{in}\;\mathrm{acidic}\;\mathrm{electrolyte}$$

(3) $$H_{2}O+e^{-}+M⇄H_{\mathrm{ads}}+{\mathrm{OH}}^{-},\;\;\mathrm{in}\;\mathrm{an}\;\;\mathrm{alkaline}\;\mathrm{electrolyte}$$

For the second step, two different pathways may occur. One is Heyrovský reaction, in which a H_2_ molecule is formed by combination of the H_ads_ on the catalyst surface, an electron and another proton (e.g. H_3_O^+^ ion), followed by desorption from the catalyst surface:

(4) $$H_{\mathrm{ads}}+H_{3}O^{+}+e^{-}\;⇄H_{2}+H_{2}O$$

The other pathway is Tafel reaction, in which a H_2_ molecule is formed by directly combining two H_ads_ and desorbs from the catalyst surface. This pathway has been confirmed in the case of Pt.

(5) $$H_{\mathrm{ads}}+H_{\mathrm{ads}}⇄H_{2}$$

Early works of this pathway indicated that the current is often correlated exponentially to the overpotential η. Given by Tafel in 1905,^16^

(6) η=a+b  log  i

A model of electrode kinetics should explain the validity of the equation above, which is known as the Tafel equation.

A Tafel plot, i.e., a plot of log *i* vs. η, is indispensable for estimating kinetic parameters. In general, there is an anodic branch with a slope of *(1–α)F/2.3RT* and a cathodic branch with a slope of *–αF/2.3RT*. As shown in Figure 1c,^[16]^ both linear segments are extrapolated to an intercept of log*i_0_*. The plots apparently deviate from linear behavior as η approaches zero, as the backward reactions can no longer be regarded as negligible. The transfer coefficient, α, and the exchange current, *i_0_*, are readily accessible from this presentation, when it can be applied. The Tafel slope, indicating the potential difference for altering the current density by 10‐fold, is often used to investigate which pathway it is involved during the HER process.

In spite of its scarcity and high cost, Pt has still been the most widely used and the state‐of‐the‐art catalyst for HER, especially in acidic conditions.^13^ A number of earth‐abundant materials have also been reported in the past decade for HER catalysts, which are almost exclusively from the transition metal elements of Co, Ni, Mo, W, Fe and Cu, and nonmetal elements of B, C, N, O, S and Se.^15^ The abundance order of these transition metal elements in the earth crust is given as: W = Mo < Co < Cu < Ni << Fe.^15^ Thus, the capability of utilizing these non‐precious metal elements, especially Fe and Ni, should be substantially important for developing large‐scale water splitting technologies. To date, a wide variety of noble‐metal‐free HER catalysts have been explored and reported for water reduction half‐reaction, including transition metals and their compounds (such as sulfides, selenides, nitrides, carbides, oxides and hydroxides), nanocarbon and their inherited composites, natural hydrogenases and artificial organometallic molecules. In this work, we will focus our discussion on those materials with 1D morphologies. Other comprehensive reviews on HER electrocatalysts can be found elsewhere.^6^, ^8^, ^11^, ^12^, ^15^, ^36^

### 2.1 Metal‐Based 1D Nanomaterials

In 2014, Sun and coworkers developed a topotactic conversion method to directly grow self‐supported nanoporous cobalt phosphide nanowire arrays on carbon cloth (designated as CoP/CC) via phosphidation of their Co(OH)F precursor.^37^ This CoP/CC nanowire array was demonstrated as a hydrogen‐evolving cathode for a wide range of pH from 0 to 14 with good stability. In the acidic condition, a low onset overpotential (38 mV) and a small Tafel slope (51 mV dec^–1^) were obtained. In the neutral and basic conditions, the onset potentials were 45 and ≈ 80 mV, and the Tafel slopes were 93 and 129 mV dec^–1^, respectively. The same research group also reported the synthesis of FeP nanowire array supported on Ti plate from its precursor,^38^ and the synthesis of self‐supported Cu_3_P nanowire arrays on porous copper foam.^39^ In these demonstrations, the non‐noble metal phosphides were synthesized by a similar low‐temperature phosphidation reaction from their corresponding (oxy)hydroxide precursors, such as β‐FeOOH and Cu(OH)_2_. A conducting substrate, such as carbon cloth, Ti plate or Cu foam, was used as the current collector to expedite the charge transport. Later, it was also found that in addition to the electrocatalytic activity, the CoP nanowire arrays also functioned as semiconductor with visible‐light absorption, enabling photocatalytic evolution of hydrogen from water under visible light.^40^ A hole‐capturing reagent, triethanolamine (TEOA) was used as a sacrificial agent. During 40 min of continuous visible‐light irradiation, the amount of H_2_ gas evolved was 66.1 µmol for the CoP nanowire arrays, while this value was increased to 160.7 µmol when a dye molecule, P_HIV_, was pre‐linked to the CoP nanowire surface. This was the first example of using CoP for photocatalytic hydrogen generation.^40^

**Figure 3:**
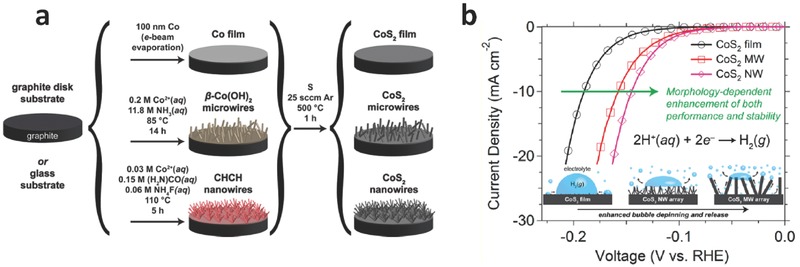
a) Schematic depictions of the preparation of a cobalt pyrite (CoS_2_) film, microwire array, or nanowire array on a graphite disk substrate. b) Electrochemical characterization of CoS_2_ film, microwire (MW) array, and nanowire (NW) array electrodes for HER electrocatalysis. Reproduced with permission.^27^ Copyright 2014, American Chemical Society.

The effect of different morphologies on their catalytic activities has also been studied for a variety of earth‐abundant HER electrocatalysts, such as MoS_2_,^41^, ^42^, ^43^ amorphous MoS_2_,^44^ WS_2_,^45^, ^46^ Ni_2_P,^47^ CoP^48^ and Ni‐Mo alloys.^49^, ^50^ In 2014, Jin and coworkers reported the synthesis of metallic cobalt pyrite (cobalt disulfide, CoS_2_) with different morphologies including films, microwires and nanowires, and showed their capabilities as a high‐activity candidate for HER.^27^ In contrast to other semiconducting pyrites like NiS_2_ or FeS_2_, CoS_2_ has a high intrinsic conductivity with a sheet resistance of ≈ 19.2 Ω sq^–1^, thus making it a promising electrocatalytic material. This stable and metallic feature of CoS_2_ is also more advantageous than other layered transition metal dichalcogenide electrocatalysts like MoS_2_ and WS_2_, which can only show high HER activity after conversion from the thermodynamically stable semiconducting phase to a metastable metallic polymorph.^27^ Different CoS_2_ morphologies were formed from thermal sulfidation of corresponding Co(OH)_2_ precursors and used for HER.^27^ It was clear that the morphologies of CoS_2_ catalysts played an important role for their HER performances, which were substantially boosted with the increase of effective electrode surface areas for microwires and nanowires (**Figure** **3**a). For CoS_2_ nanowires, a geometric current density of –10 mA cm^–2^ was obtained at an overpotential as low as –145 mV vs. reversible hydrogen electrode (RHE) (Figure 3b). In addition, both the micro‐ and nanowires provided a synergistic effect by enhancing the evolution of gas bubble from the electrode surface. With CoS_2_ films, large bubbles tended to be trapped at the electrode surface, thus reducing the catalytically‐active sites.^27^ In contrast, the 1D micro‐ and nanostructures provide high surface area and large surface curvature, air bubbles attached to these 1D micro‐ and nanostructures have much higher surface energy, and thus tend to be released from the surface. This phenomenon is similar to air bubbles at the microstructured surface of a lotus leaf.^51^ As a result, more rapid liberation of catalytically‐active sites is achieved by faster release of bubble, thus benefiting for further contact of electrolytes on the catalyst/electrode surface. Later, the same research group reported a type of ternary pyrite‐type cobalt phosphosulphide (CoPS) nanowires as a highly efficient catalyst for both electrochemical and photoelectrochemical hydrogen evolution.^52^ By combining with a theoretical study, it was shown that the CoPS nanowire electrodes presented a geometrical current density of 10 mA cm^–2^ at a low overpotential of 48 mV vs. RHE. When integrated with n^+^‐p‐p^+^ silicon micropyramids for photoabsorption and conversion, this hybrid catalyst/electrode enabled a photocurrent density of 35 mA cm^–2^ at 0 V vs. RHE, and onset photovoltages as high as 450 mV vs. RHE, which marked the most efficient solar‐driven hydrogen generation from earth‐abundant materials.

By a similar design concept, our group demonstrated the conversion of spinel‐type nickel cobalt oxide (NiCo_2_O_4_) nanowires into pyrite‐type nickel cobalt sulfide (Ni_0.33_Co_0.67_S_2_) nanowires, via a thermal sulfidation process.^35^ The obtained Ni_0.33_Co_0.67_S_2_ nanowires presented 1D morphology and expedited charge transport capability, and thus served as an stable and efficient HER catalyst for a wide pH range. Under the acidic, neutral and basic electrolytes, the onset potentials were measured as low as –65, –39 and –50 mV vs. RHE, and Tafel slopes were 44, 68 and 118 mV dec^–1^, respectively, which were much better than individual CoS_2_ or NiS_2_ nanostructures under similar conditions. In addition to phosphides and sulfides, carbides have also been synthesized in 1D structures for HER.^53^ For example, nanoporous Mo_2_C nanowire arrays were synthesized by pyrolysis of a MoO_x_/amine hybrid precursor under an inert atmosphere.^54^ Due to the close contact of MoO_x_ and amine molecules, a quasi‐homogeneous reaction environment was created, which facilitated the formation of nanocrystallites, abundant nanoporosity, and large active surface area, thus leading to excellent HER activity.

### 2.2 Incorporation of Metal‐Based Nanomaterials on 1D Substrates

**Figure 4:**
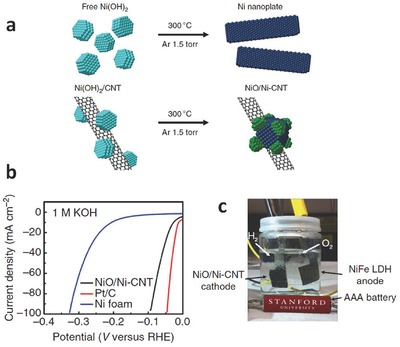
a) Schematic illustration of the structural difference between CNT hybrid and pure nanoparticle. b) Linear sweep voltametry of NiO/Ni‐CNT, Pt/C deposited on Ni foam and pure Ni foam at a scan rate of 1 mV s^–1^ under the loading of 8 mg cm^–2^ in 1 M KOH. c) Demonstration of water‐splitting device powered by an AAA battery with a nominal voltage of 1.5 V. Reproduced with permission.^56^ Copyright 2014, Nature Publishing Group.

The direct growth or attachment of the effect metal phosphides, sulfides, carbides or selenides onto nanoscale structure surface has also been regarded as an important approach for making the 1D catalyst/electrode interface. Among many nanostructured electrode candidates, carbon nanotubes (CNTs) are the most recognized one, due to their convenient and large‐scale synthesis, high intrinsic conductivity and environmental benignity.^55^ Nitrogen doping has also been interrogated to further increase the conductivity and electrocatalytic activity of CNTs, due to the comparatively higher electron negativity of nitrogen atoms than carbon.^12^ In 2014, Dai and coworkers reported the formation of nanoscale NiO/Ni heterostructures on the CNT sidewalls as an active and stable HER catalyst.^56^ The NiO/Ni heterostructure was fabricated serendipitously by thermal annealing, resulting in partially reduced Ni(OH)_2_ nanoparticles. The CNTs were partially oxidized and impeded the complete reduction and Ostwald ripening of Ni species into the less HER active Ni phase (**Figure** **4**a). Compared to NiO/CNT and Ni/CNT samples, the NiO/Ni‐CNT catalyst exhibited a clearly better HER activity with a lower overpotential and a higher current density (Figure 4b), thus indicating a synergistic effect between NiO and Ni. The electrocatalytic activity was close to that of Pt under similar conditions. When this NiO/Ni‐CNT was coupled with a NiFe‐layered double hydroxide (NiFe LDH) water oxidation catalyst and used as cathode and anode, respectively, the water electrolyzer achieved a current density of 20 mA cm^−2^ at an applied potential of 1.5 V by a single‐cell AAA alkaline battery (Figure 4c).

In addition to Ni, the Co‐based, Mo‐based and Fe‐based materials have also been investigated by incorporating on the surface of CNTs. In 2014, Asefa and coworkers reported the synthesis of Co‐embedded, nitrogen‐doped CNTs, using a thermal treatment of Co^2+^‐incubated CNTs from inexpensive dicyandiamide and CoCl_2_ as starting materials.^57^ It was found that these Co‐embedded N‐doped CNTs functioned well under acidic, neutral or basic electrolytes with a HER activity close to Pt. Structural analysis revealed that this good activity was mainly attributed to the nitrogen dopants and concomitant structural defects. Similarly, amorphous molybdenum sulfide (MoS_x_) layer (≈2 nm thick) was formed by decomposition of precursors onto N‐doped CNT forest surface.^58^ An onset potential of 75 mV and a small overpotential of 110 mV for a current density of 10 mA cm^–2^ were achieved, which represented the highest HER performance for MoS_x_‐based materials at that time. Deng et al. developed a method of encapsulating Fe, Co and Fe/Co alloy into nitrogen‐doped CNTs by using a chemical vapor deposition (CVD) approach,^59^ which exhibited an excellent stability and a high activity with ≈ 70 mV onset overpotential vs. RHE, close to that of commercial 40% Pt/C catalyst. In another study, using dicyanodiamine as precursor and Co_3_O_4_ nanowire array as catalyst, the Sun group reported the direct chemical vapor deposition (CVD) synthesis of a film of interconnected Co‐entrapped, nitrogen‐doped CNTs on carbon cloth.^60^ The dense CNT networks provided a high catalyst loading and strong coupling of the catalyst, and the obtained catalyst showed a high hydrogen‐evolving activity and durability over the whole pH range.

Similar to the aforementioned conversion of free‐standing oxides or (oxy)hydroxides, the in situ conversion of these materials grown on CNTs into corresponding sulfides, phosphides, and so on, can also processed in a similar manner. For instance, CoP nanocrystals decorated on CNTs were synthesized by the low‐temperature phosphidation of a Co_3_O_4_/CNT precursor.^61^ Co‐doped iron pyrite nanosheets were interfaced with CNTs (Fe_1–x_Co_x_S_2_/CNT hybrid) by solution reaction of Fe salt, Co salt and thioacetamide, followed by thermal treatment.^62^ A strong dependence of the Co doping ratio on the HER activity of the Fe_1–x_Co_x_S_2_/CNT hybrid catalyst was found. For 10% of Co doping with a high catalyst loading (≈7 mg cm^–2^), the Fe_0.9_Co_0.1_S_2_/CNT hybrid presented a lowest overpotential of ≈0.12 V at a current density of 20 mA cm^–2^, and a Tafel slope of 46 mV dec^–1^.

### 2.3 Metal‐Free 1D Carbonaceous Nanomaterials

**Figure 5:**
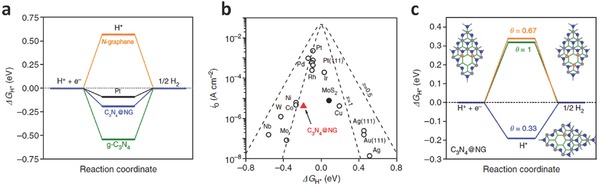
a) The calculated free‐energy diagram of HER at the equilibrium potential for three metal‐free catalysts and Pt reference. b) Volcano plots of exchange current density as a function of the ΔG_H*_ for newly developed C_3_N_4_@NG (red triangle), common metal catalysts as well as a typical nanostructured MoS_2_ catalyst. c) Free‐energy diagram of HER on the surface of C_3_N_4_@NG under different H* coverage (1/3, 2/3 and 1 with the molecular configurations shown as insets) conditions. Reproduced with permission.^65^ Copyright 2014, Nature Publishing Group.

In spite of these progresses, the use of metal ions in these catalysts still encounters the possibility of metal ion dissolution and release, thereby reducing the catalytic activity and increasing the environmental impact. The development of metal‐free catalysts has also become an important focus recently, mainly for the carbon‐based materials. For instance, it was first reported in 2014 that pristine CNTs were active by acidic oxidation to achieve enhanced electrochemical reduction capability of converting protons to hydrogen,^63^ although the strong acidic oxidation may also deteriorate the electrical conductivity and the catalytic activity. Likewise, carbon nitride (C_3_N_4_) can also be synthesized to show similar HER catalytic activity. For example, using the hydrogen‐bonded cyanuric acid melamine supramolecular complex as precursor, 1D C_3_N_4_ nanorod were synthesized on a variety of different substrates, due to the interaction of free hydroxyl and amine groups of the supramolecular complex precursor.^64^ One early bench mark progress in this field was reported by the Qiao group, who reported the preparation of graphitic‐C_3_N_4_ coupled with N‐doped graphene (C_3_N_4_@NG).^65^ This metal‐free hybrid catalyst exhibited an unexpectedly high HER activity that was comparable to some of the well‐developed metallic catalyst. Density functional theory calculations showed that the free‐energy changes of H* adsorption and H_2_ desorption from C_3_N_4_@NG was closer to the level of Pt than those of N‐doped graphene and graphitic‐C_3_N_4_ (**Figure** **5**a). The Δ*G*_H*_ of the developed C_3_N_4_@NG was also plotted together with common metal catalysts in the volcano plot (Figure 5b), and the free energy diagram for different surface coverage of H* on C_3_N_4_@NG was also calculated (Figure 5c). Together with the experimental approaches, this study revealed that the unconventional electrocatalytic property was originated from an intrinsic chemical and electronic coupling, which synergistically enhanced the proton adsorption and reduction kinetics. Furthermore, theoretical approaches have robustly been used to interrogate the mechanism of the metal‐free catalytic process, which revealed that the Volmer‐Heyrovsky step is the predominant HER mechanism while the Heyrovsky step is the rate‐determining for CNTs.^66^, ^67^

## 3 OER

Likewise, the oxygen evolution can take place in either acidic or alkaline conditions, with the corresponding half reactions as:

(7) $$O_{2}+4H^{+}+4e^{-}⇄{\mathrm{2H}}_{2}O,\;\;\mathrm{in}\;\mathrm{acidic}\;\mathrm{electrolyte}$$

(8) $$O_{2}+2H_{2}O+4e^{-}⇄\;{\mathrm{4OH}}^{-},\;\;\mathrm{in}\;\;\mathrm{an}\;\;\mathrm{alkaline}\;\;\mathrm{electrolyte}$$

This 4‐electron half‐reaction involves multiple reaction intermediates (i.e., OOH*, O* and OH*) in the mechanism, either in acidic or alkaline conditions.^18^ Compared to HER, the water oxidation half‐reaction (OER) is much more sluggish in kinetics. Generally, it is believed that the mechanism is attributed to the interaction of oxygen atom with the *d*‐electrons of the transition metal ions.^8^, ^18^ The best reported OER performances are still marked by the noble metal oxides such as RuO_2_ and IrO_2_, with overpotentials of as low as 140 mV for RuO_2_ in alkaline conditions.^68^, ^69^ To date, for earth‐abundant metal‐based materials, most of the non‐noble‐metal oxides and hydroxides reported for OER catalysts are focused on those of Co, Ni, Mn, Fe, Mo and W.^8^, ^18^, ^70^

### 3.1 Metal‐based 1D Nanomaterials

It has been long known that Co_3_O_4_ is an ideal OER catalyst with high efficiency and good corrosion stability,^71^ but previous studies of these Co‐based oxides for OER were predominantly in the form of thin films or agglomerated particles. One of the earliest examples of using 1D Co_3_O_4_ nanowires for OER was reported in 2010,^26^ in which the synthesis of mesoporous Ni_x_Co_3–x_O_4_ nanowire arrays was first achieved on the surface of Ti foils via an ammonia evaporation induced growth. Spectroscopic characterizations indicated that the Ni element had an uneven distribution and was concentrated on the nanowire surface. Compared to Co_3_O_4_ nanowires without Ni doping, this Ni doping allowed for the enhancement of the OER activity, with enhanced electrical conductivity and double layer capacitance. Other elemental dopings of the Co_3_O_4_ have also been reported. For instance, by co‐deposition of both Zn and Co molecular precursors on Ti foils followed by thermal conversion, 1D Zn_x_Co_3–x_O_4_ branched nanostructures were constructed with small secondary nanoneedles directly grown from primary rhombus‐shaped nanopillar arrays.^72^ A small overpotential of ≈ 0.32 V was required for this Zn_x_Co_3–x_O_4_ nanostructure to achieve 10 mA cm^–2^, with a Tafel slope of 51 mV dec^–1^, substantially exceeding those of pristine Co_3_O_4_ NWs.

Besides elemental doping, the tuning of the oxygen vacancies of those oxides has also been demonstrated as an effective means, as the oxygen vacancies are known as the charge carriers of many oxides such as Co_3_O_4_.^73^ Previously, the thermal annealing in a reducing environment such as hydrogen gas was demonstrated to enhance the electrical performances of TiO_2_ and other metal oxides,^74^, ^75^, ^76^ while this hydrogen annealing is not energy efficient or safe, and not applicable especially for the thermally instable materials. In 2014, our group reported a mild, solution‐based approach to chemically reduce Co_3_O_4_ nanowires into their reducing forms with NaBH_4_.^73^ In that work, mesoporous Co_3_O_4_ nanowires were first synthesized with a surface area of 58 m^2^ g^–1^ and a mean mesopore size of 6–8 nm. Afterwards, these nanowires were reduced in a NaBH_4_ solution at room temperature. Although no significant differences in the morphology or structure were observed, the spectroscopic measurements indicated the increase of oxygen vacancies inside the pristine Co_3_O_4_ crystal structures. Compared to the pristine counterparts, the chemically reduced Co_3_O_4_ nanowires presented a much lower onset potential of 1.52 vs. RHE, and a much larger current density of 13.1 mA cm^–2^ at 1.65 V vs. RHE. Density functional theory calculations suggested that the existence of oxygen vacancies inside these oxides result in the formation of new gap states, where the electrons previously associated with the Co‐O bonds tend to be delocalized, leading to much higher OER activity.^73^

In addition to Co‐based oxides and (oxy)hydroxides, other transition metal compounds have also been developed for OER. For instance, manganese oxides with different structures, including α‐, β‐, δ‐MnO_2_ and amorphous) were synthesized and compared.^77^ The structures of different MnO_2_ depend on the connectivity between the [MnO_6_] units via sharing corners or edges, with δ‐MnO_2_ as layered structures and α‐, β‐MnO_2_ as 1D structures. It was found that the OER activity of these MnO_2_ catalysts in an alkaline media was strongly dependent on the crystallographic structures, with an order of α‐MnO_2_ > amorphous > β‐MnO_2_ > δ‐MnO_2_. An overpotential of 450 mV was required for α‐MnO_2_ to present a current density of 10 mA cm^–2^, compared to a 380 mV overpotential required by an Ir/C catalyst. Meanwhile, at a constant current density of 5 mA cm^–2^, a 3‐h stability was achieved by α‐MnO_2_, which was further extended to ≈ 8 h when adding with Ni dopants. In another example, Zhang and coworkers reported the synthesis of Ni_3_S_2_ nanorods on Ni foam by a simple hydrothermal reaction.^70^ Spectroscopic measurements revealed that rich and unconventional oxidation states of Ni (Ni^0^, Ni^+^ and Ni^2+^) existed in the Ni_3_S_2_/Ni composite, owing to the synergetic chemical coupling effects among Ni_3_S_2_ nanorods, NiO layer and the Ni foam support, thus leading to an ultra‐low overpotential. In a 0.1 M KOH solution, an overpotential of as low as ≈ 187 mV was required to achieve a current density of 10 mA cm^–2^, much lower than that of other transition metal oxides or 20 wt% Pt/C. This value was also comparable to that of RuO_2_, marking the best OER performance among the non‐noble metal inorganic electrocatalyst at that time.

**Figure 6:**
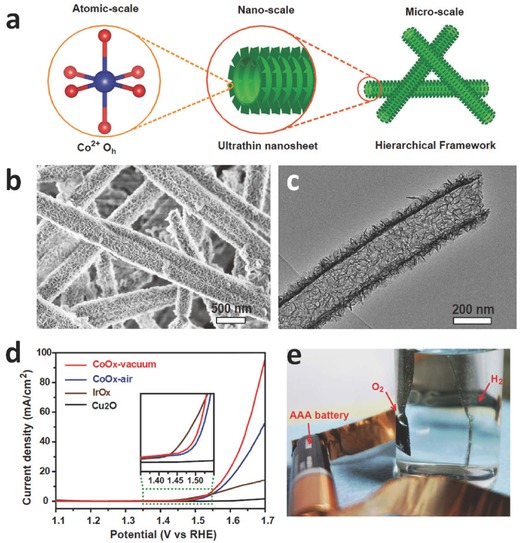
a) Schematic illustration of design of the hierarchical CoO_x_ nanosheet/nanotube structures with multiple scale optimizations. b) SEM and c) TEM images of the hierarchical CoO_x_ nanosheet/nanotube structures. d) Water oxidation current of the CoO_x_‐vacuum (red curve), CoO_x_‐air (blue curve), IrO_x_ (brown curve), and CuO nanowire (black curve) at 5 mV s^−1^. Inset: the zoom‐in plot of the onset potential region. e) Demonstration of water splitting cell powered by a 1.5‐V AAA battery. The CoO_x_ catalyst was loaded on Ni foam with a loading mass of 4 mg cm^–2^ as an anode and a Pt‐mesh was used as a cathode. Reproduced with permission.^78^

Although 1D nanostructures provide efficient charge transport channels and abundant space between their neighbors, the surface areas of pristine 1D nanostructures may still be lower compared to 0‐ or 2‐dimentional nanomaterials. Inspired by the heterostructure of plant leaves, our group developed a 2D/1D CoO_x_ heterostructure by a solution‐based cation exchange process, which was composed by ultrathin CoO_x_ nanosheets that were further assembled into a nanotube structure.^78^ This 2D/1D CoO_x_ heterostructure enabled the optimization of structure and OER activity over different length scales (**Figure** **6**a). At the atomic scale, the Co^2+^ presented an octahedral electronic structure that is beneficial for OER. At the nanoscale, the self‐assembly of high‐density, ultrathin CoO_x_ nanosheets precluded the aggregation or accumulation of sheet thickness, thus leading to an ultrahigh surface area of 371 m^2^ g^–1^ ever obtained for CoO_x_ (Figure 6b, c). At the microscale, these few‐layered CoO_x_ nanosheets were assembled into a large 3D, porous framework of nanotubes, which allowed for efficient charge and ion transport. Using this 2D/1D CoO_x_ heterostructure as an OER catalyst, a low onset potential of ≈1.46 V vs. RHE was achieved, and the current density was increased to 51.2 mA cm^–2^ at 1.65 V vs. RHE (Figure 6d, e). Furthermore, by coupling this 2D/1D CoO_x_ heterostructure and a Pt‐mesh as the anode and cathode, respectively, a full water splitting electrolyzer was demonstrated using a single 1.5 V AAA alkaline battery, with a stable current density of ≈1 mA cm^–2^ for over 2 h.^78^ Later, our group further developed the synthetic process by extending this growth strategy to a variety of 1D transition metal oxide structures such as MnO_x_, ZnO_x_, NiO_x_, FeO_x_,^79^ and mixed Co‐Ni hydroxides, as well as direct growth of these 1D nanostructures on conductive substrates.^80^ These transition metal‐based oxides and (oxy)hydroxides featured excellent OER activities, which were attributed to their abundant electrochemically reactive surface sites, 1D morphology for fast charge transport, and direct electrical contact to the underlying current collectors.

### 3.2 Incorporation of Metal‐Based Nanomaterials on 1D Substrates

In addition to 1D morphologies of metal compounds, these metal‐based catalysts can also be loaded onto different types of 1D conducting substrates to obtain the optimal performance. This design strategy is similar to the aforementioned incorporation of these metal‐based nanomaterials on 1D substrates for HER catalysts. Thus, here we only specify some representative examples. For instance, a conducting network of copper nanowires was used to be electrodeposited with a conformal layer of nickel or cobalt around the nanowires, which was used as water oxidation catalyst.^81^ A unique sandwich‐like, 3D coaxial structure of Ni nanotube array coated with Ni and Co co‐hydroxide nanosheets was demonstrated,^82^ which featured both high surface area and enhanced electron transport. Nitrogen‐doped CNTs were also used to incorporate with spinel Mn‐Co oxide nanoparticles by oxidative thermal scission.^83^ During this process, the N‐doped CNTs were ruptured, and the partially embedded Mn and Co nanoparticle catalysts were oxidized, with intimate contact to the graphene walls of CNTs. Recently, our group also demonstrated the conversion of a biomass, egg‐shell membrane, into 3D grid‐like fibrous carbon microstructures with abundant macropores, which were used for the growth of NiCo_2_O_4_ nanowire arrays for efficient water oxidation.^84^

### 3.3 Metal‐Free 1D Nanomaterials

**Figure 7:**
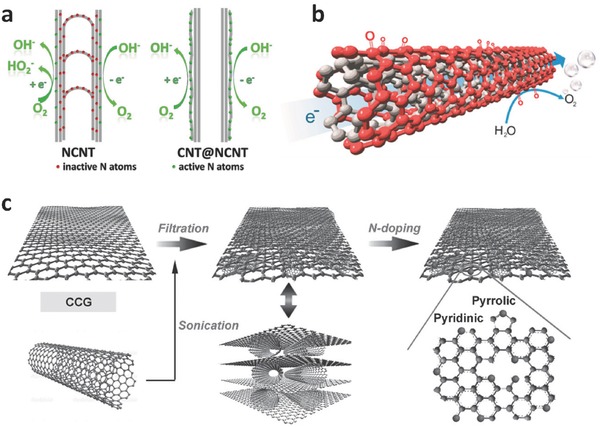
a) Schematic of the full exposure of active sites on the surface: NCNTs with bulk doping of nitrogen atoms, while CNT@NCNT coaxial nanocables with surface enriched nitrogen for OER. Reproduced with permission.^85^ b) Schematic of the generation of oxygen‐containing functional groups on the surface of CNTs. Reproduced with permission.^86^ Copyright 2015, American Chemical Society. c) Schematic of the layer‐by‐layer synthetic process of NG‐CNT. Reproduced with permission.^55^

Similar to HER, the metal‐free OER catalyst has also been explored recently, in which CNTs or N‐doped CNTs are the most extensively studied for 1D structures. In 2014, Tian et al. reported the synthesis of N‐doped carbon coaxial nanocables, in which pristine CNTs and N‐doped carbon layers were served as the core and shell (**Figure** **7**a).^85^ This coaxial 1D nanostructure allowed the enrichment of nitrogen atoms on the surface as well as the intact inner carbon walls, rendering a high electrical conductivity of 3.3 S cm^–1^ and a much enhanced OER activity for the catalyst. It was also reported that the surface of multiwalled CNTs could be tuned into an OER catalyst by mild surface oxidation, hydrothermal annealing and electrochemical activation (Figure 7b).^86^ The catalytic mechanism was ascribed to the generation of oxygen‐containing functional groups such as ketonic C = O on the outer walls of CNTs, which altered the electronic structures of adjacent carbon atoms and facilitated the adsorption of OER intermediates.

In addition to single carbon structures, Qiao and coworkers reported the dual doping of nitrogen and oxygen on graphene‐CNT hydrogel film,^55^ via a layer‐by‐layer assembly of CNTs and graphene (with intrinsic oxygen impurities) through a simple filtration method, followed by annealing in ammonic for nitrogen doping (Figure 7c). The high OER activity of this hybrid catalyst was attributed to the dual active sites originating from the chemically converted CNTs and graphene. Recently, our group reported the growth of a heterostructure of C_3_N_4_/CNTs/carbon fiber.^87^ The OER performance was owing to the high nitrogen content of C_3_N_4_ and the enhanced charge transport capability of the 1D/3D hierarchical carbon networks.

## 4 Bifunctional HER/OER Catalyst

As described above, a number of earth‐abundant non‐noble materials have been investigated and developed in the past years as separate HER and OER catalysts, with substantial progresses of improving both the catalytic performances and long‐time stability. Nonetheless, the HER catalysts such as transition metal sulfides, selenides and phosphides still function the best in acidic media, while the OER catalysts generally need to be used in alkaline conditions to avoid material dissolution.^10^ The use of separate electrolytes (as well as separation films) within the same electrolyzer inevitably reduces the cell efficiency while at the same time increases the fabrication cost. Thus, the capability of preparing single materials that allow for both HER and OER in the same electrolyte represents a significant merit, not only for optimizing the conversion efficiency but also for large‐scale and cost‐effective deployment. In the past few years, a quick increase of research focus has been realized and dedicated to this field, with the efforts generally divided into two directions: (1) developing homologous material pairs that can be converted for HER and OER, respectively; and (2) exploring new materials/structures that are efficient for both HER and OER simultaneously.

### 4.1 Homologous Material Pairs

One of the earliest demonstrations of the non‐noble‐metal bifunctional HER/OER electrocatalysts, although not in 1D morphologies, was reported by the Artero group in 2012.^88^ In their work, a nanoparticulate material, namely H_2_‐CoCat, was electrochemically synthesized from cobalt salts in a phosphate buffer solution. This new material was composed of metallic cobalt coated with a cobalt‐oxo/hydroxo‐phosphate layer, which allowed for hydrogen production from neutral buffer. Further electrochemical equilibration converted this H_2_‐CoCat into a new form of amorphous cobalt oxide film, namely O_2_‐CoCat that enabled O_2_ evolution. It was found that the switching between these two material forms was reversible, which was also well correlated with the conversion of the morphologies at the electrode surface and two types of non‐metallic atomic structures. Nonetheless, the catalytic properties of these early works were still far from optimum. Later, using a similar synthetic approach, Yu et al. demonstrated that by direct electrodepositing a Ni‐based bifunctional material on 1D multiwalled CNTs, the performance of the electrocatalytic activity was much improved.^89^ Spectroscopic studies revealed that the content of oxygen in the O_2_‐NiO_x_‐CNT film (i.e., the OER catalyst) was higher than that in the H_2_‐NiO_x_‐CNT film (i.e., the HER catalyst). The Faradaic efficiencies for hydrogen and oxygen production were over 95%.

The chemical conversion between OER and HER catalysts using sulfidation or phosphidation has also been investigated. For example, Peng et al. reported the conversion of spinel‐type NiCo_2_O_4_ nanowires to pyrite‐type Ni_0.33_Co_0.67_S_2_ nanowires by sulfurization, which functioned as HER and OER catalysts, respectively.^35^ A water splitting electrolyzer using these all‐nanowire‐based catalysts achieved a current density of 5 mA cm^–2^ at 1.65 V. Similarly, Li et al. demonstrated the conversion of Co‐Ni hydroxide nanowires into Co‐Ni nitride nanowires by nitridation.^80^ By using these Co‐Ni hydroxide and nitride nanowires as anode and cathode, a current density of > 10 mA cm^–2^ was obtained at 1.65 V. Although these water splitting experiments were carried out in the same electrolyte, it still requires extra steps for conversion between two material forms and thus increases complexity and cost.

### 4.2 Single Nanostructure for HER and OER

The single composite of bifunctional electrocatalyst was first studied with metal components. In 2014, the Bao group reported the preparation of cobalt nanoparticles encapsulated in N‐doped carbon (designated as Co@N‐C), which displayed good HER performance in a wide pH range and OER performance in alkaline solution.^90^ The applied potentials for Co@N‐C in alkaline condition (1 M KOH) for HER and OER were measured as 0.33 and 1.63 V, in order to achieve current densities of 10 mA cm^–2^. These results were better than iron nanoparticles encapsulated in N‐doped CNTs with similar nitrogen doping level. For the full electrolyzer using this Co@N‐C as cathode and anode, a current density of ≈ 40 mA cm^–2^ was obtained at an applied bias of 1.7 V.

In addition to serve as a conducting substrate, CNTs were also explored as templates for synthesize 1D nanostructures. The Leonard group prepared 1D nanocrystalline Mo_2_C using multiwalled CNTs as templates.^91^ Although it was known that Mo_2_C could serve as a HER catalyst, its capability for OER had not been reported before. Using four different synthetic methods, the authors examined the electrocatalytic activities of these four products for HER in acid and alkaline electrolytes as well as OER in alkaline media. It was found out that 1D Mo_2_C nanostructures prepared with CNT templates presented the best performance among these four products, suggesting the virtues of 1D structures. Later, Tian et al. used a template‐assisted electrodeposition method to synthesize a NiMo‐alloy hollow nanorod array.^92^ To obtain 10 mA cm^–2^ current densities for HER and OER, overpotentials of 92 and 310 mV were required for the NiMo nanorod array, respectively. At an applied voltage of 1.64 V, this bifunctional electrocatalyst enabled an alkaline electrolyzer with 10 mA cm^–2^.

**Figure 8:**
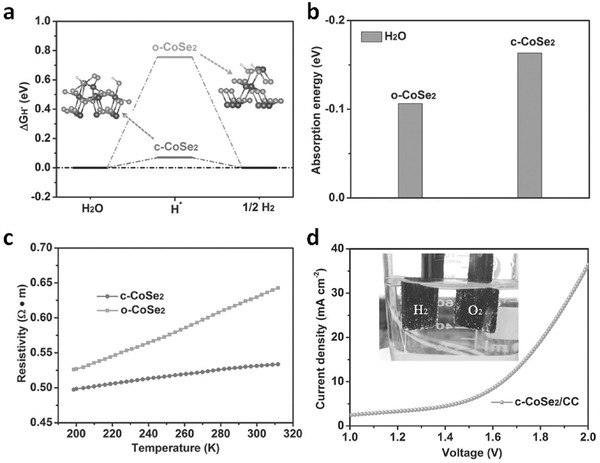
a) Calculated HER free‐energy change and b) water adsorption energy for the *o*‐CoSe_2_ and *c*‐CoSe_2_ products. c) Temperature‐dependent resistivity of simple *o*‐CoSe_2_ and *c*‐CoSe_2_ products. d) Polarization curve of water electrolysis using *c*‐CoSe_2_/carbon cloth as both HER and OER electrocatalysts in a two‐electrode configuration with a scan rate of 10 mV s^−1^. Inset: Optical photograph showing the generation of H_2_ and O_2_ bubbles on the *c*‐CoSe_2_/carbon cloth electrode. Reproduced with permission.^93^

Chalcogenides are the most extensively approached targets for bifunctional electrocatalyst. For example, CoSe_2_ is a well‐known earth‐abundant electrocatalyst, with two possible crystalline phases, the orthorhombic macarsite‐type (*o*‐CoSe_2_) and the cubic pyrite‐type (*c*‐CoSe_2_) structures. However, previous studies only focused on the acidic HER activity or the alkaline OER activity of CoSe_2_. Wu, Xie and coworkers recently reported the phase transformation strategy and controlled synthesis of these two different CoSe_2_ phases.^93^ Density functional theory calculations showed the HER free energy change and water adsorption energy for the *o*‐CoSe_2_ and *c*‐CoSe_2_ products (**Figure** **8**a). It was found out that under alkaline conditions, the *c*‐CoSe_2_ catalyst exhibited optimal water adsorption energy (Figure 8b), higher electrical conductivity (Figure 8c), and quicker kinetics and efficiency transforming the adsorbed hydrogen into H_2_, thus leading to better HER electrocatalytic activity over *o*‐CoSe_2_. An alkaline water splitting electrolyzer based on this *c*‐CoSe_2_ catalyst showed a current density of 10 mA cm^–2^ under 1.63 V, with excellent stability over 10,000 seconds (Figure 8d).

In 2015, the Sun group demonstrated the synthesis of NiSe nanowire film on nickel foam by a in situ hydrothermal method, using nickel foam and NaHSe as Ni and Se sources, respectively.^94^ This NiSe nanowire grown on Ni foam behaved as a 3D electrode for both HER and OER in strongly alkaline media, with superior catalytic activity and durability. A cell voltage of 1.63 V was needed to drive the water electrolyzer to achieve 10 mA cm^–2^. The same group also reported the conversion of NiCo_2_O_4_ nanowires on carbon cloth into NiCo_2_S_4_ nanowires,^95^ where the latter enabled 10 mA cm^–2^ current density for full water splitting at a cell voltage of 1.68 V, which was 300 mV lower than that for NiCo_2_O_4_ under the same condition. Sivanantham et al. showed the direct growth of 1D NiCo_2_S_4_ nanowire arrays on 3D Ni foam, which delivered 10 mA cm^–2^ under a cell voltage of 1.63 V in an alkaline electrolyzer.^96^ Other sulfides, selenides, phosphides and nitrides, including Ni‐doped CoS_2_ nanowires,^97^ Ni_3_Se_2_ nanoforests,^98^ mesoporous CoP nanorod arrays,^99^ hierarchical TiN@Ni_3_N nanowire arrays,^100^ have also been reported.

**Figure 9:**
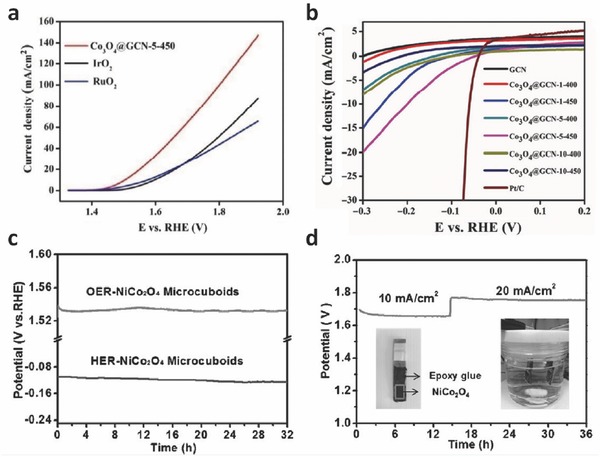
a) LSV curves of the Co_3_O_4_@GCN hybrid, IrO_2_, and RuO_2_ at 1,600 rpm in 1 M KOH for OER. b) LSV curves of all samples and Pt/C at 1,600 rpm in 0.5 M H_2_SO_4_ for HER. Reproduced with permission.^101^ Copyright 2015, Springer. c) Galvanostatic measurement of OER and HER by NiCo_2_O_4_ hollow microcuboids in 1 M NaOH at a current density of 10 and –10 mA cm^–2^, respectively. d) Overall water‐splitting characteristics in a two‐electrode configuration at current densities of 10 and 20 mA cm^–2^. Inset in (b) shows optical image of NiCo_2_O_4_ electrode and overall water‐splitting device. Reproduced with permission.^102^

Although oxides are typically known with good OER activity but not for HER, their potential for hydrogen evolution has also been investigated recently. The combination of oxides with graphitic carbon materials has been demonstrated as bifunctional catalysts, as the metal oxides and graphitic carbon materials can contribute each own strength for OER and HER, respectively, as previously reported by Co‐Mn complex oxide superlattice coated by N‐doped carbon conjugate.^23^ Recently, Tahir et al. reported a facile, large‐scale chemical synthesis of Co_3_O_4_ embedded 1D tubular nanostructures of graphitic carbon nitride, which exhibited strong synergistic effect between Co_3_O_4_ and graphitic carbon nitride.^101^ This hybrid conjugate showed the lowest overpotential of 0.12 V for OER (**Figure** **9**a), even smaller than RuO_2_ (0.14 V) and IrO_2_ (0.16 V). For HER, the composite presented a small onset of –0.03 V in 0.5 M H_2_SO_4_ (Figure 9b), close to that of commercial Pt/C (–0.01 V). As the onset potential of plain graphitic carbon nitride was –0.27 V, it was concluded that the co‐existence of Co_3_O_4_ was beneficial for the HER performance. However, the HER and OER experiments in this work were not carried out under the same pH conditions, and thus the full water splitting was not demonstrated. The mechanism of the synergistic effect between Co_3_O_4_ and graphitic carbon nitride was still not fully understood. More recently, Gao et al. demonstrated that an unconventional, hierarchical NiCo_2_O_4_ hollow microcuboids, consisting of 1D nanowires, were synthesized and used as good HER catalyst.^102^ In 1 M KOH solution, the onset potential for HER was measured as –50 mV. The overpotentials for driving cathodic current densities of 10 and 100 mA cm–^−2^ were –110 and –245 mV, respectively (Figure 9c). For the overall water splitting, the current densities reached 10 and 20 mA cm^–2^ at applied potentials of 1.65 and 1.74 V, respectively (Figure 9d). These results were much better than those of NiCo_2_O_4_ with microflower morphology, thus suggesting the importance of structural effect on water splitting performance.

## 5 Conclusion

In the past decade, the research community has been witnessing a substantial burst of interest and effort in searching new electrocatalysts for water splitting, with exquisite developments of controlling the synthesis of new materials/composites and the fabrication of new water splitting devices/systems. Due to the well‐known limitation in using noble metal‐based materials, it has been widely recognized that highly active and robust electrocatalysts, made of earth‐abundant materials with low cost and good sustainability, are indispensable for realizing scalable water splitting with high efficiency and excellent stability. In addition, it has also been well envisioned that nanoscale materials such as 1D nanostructures can bring significant benefits for achieving high apparent electrocatalytic activity for practical applications, which are generally attributed to the following features. First, the electrochemically active surface area of these 1D nanostructures can be substantially enhanced compared to their thin film or conjugate counterparts, resulting in more surface reaction capability. Second, the electrical conductivities of these 1D nanostructures can be well optimized due to their increased crystalline and reduced grain boundaries, thus enhancing the charge transport and transfer processes. Further introducing of additional conducing agents/substrates such as metal or carbon into the composite can further expedite the charge transport along these materials. Third, due to the inter‐spacing between their adjacent neighbors, the mass transfer of electroactive reactants or products can be much enhanced, especially towards the deep portion of the aligned 1D nanostructure arrays, which are clearly advantageous than their bulk or thin film counterparts.

This review has introduced the recent progresses in designing and developing 1D earth‐abundant nanomaterials/nanostructures as active HER or OER catalysts, with a brief summary of their preparation methods and electrocatalytic performances for water splitting. In spite of these achievements in developing low‐cost catalysts, the research and investigation are still at an early stage. Several significant challenges exist, for both 1D nanomaterials and other structures, and need to be solved in order to reach the next‐stage milestones for catalytic water splitting with higher efficiency and stability.

First of all, the mechanism of water splitting with electrocatalysts needs to be further elucidated. The understanding of the mechanism of different HER, OER and bifunctional catalysts is not only important from fundamental science point of view, but also suggests potential directions for optimization of current catalyst behaviors as well as further rational design/synthesis of new materials for catalyst candidates. Although substantial studies have been carried out in investigating the structure‐function inter‐relationship of various catalysts with corresponding activity and stability, there is still lack of in‐depth mechanistic investigation on an atomic level. The HER process for Pt under acidic conditions is relatively understood, but remains quite ambiguous under basic conditions especially for those composite materials. For OER and bifunctional catalysts, the knowledge of their working mechanisms towards water splitting is still preliminary. In particular, the fundamental studies on the electrochemical properties, electron‐transfer behavior and multiple‐electron coupled reaction mechanism are quite limited. Thus, a close integration of both advanced in situ characterization techniques and theoretical simulations is necessary to clarify the catalytic mechanism and advance the field.

Second, the development of new electrocatalytic materials nowadays has still largely depended on pre‐requisite knowledge of existing materials and trial‐and‐errors, but very few materials have been discovered based the rational design and fundamental understanding the catalytic mechanism and the targeted electrochemical reactions.^17^ Most of the works in this field have been focused on the observed electrochemical properties of these catalysts such as values of overpotentials and Tafel slopes, but the fundamental nature of these properties has largely been missing, which limit the capability of realizing new materials with significantly improved performances. For example, the bifunctional earth‐abundant catalysts reported to date are all based on alkaline electrolyzers, which require additional gas separation steps. The bifunctional catalysts that can be stable in acidic conditions have not been discovered yet. The in‐depth understanding of these features can surely expedite the material screening process, and may further point to new elements or composites that have not been studied for this application. In addition, although based on some simplified models, the DFT calculations are capable of providing a reasonably high level of accuracy to evaluate experimental data and predict potential directions.

Moreover, the comparison and leverage of different works are still difficult. For instance, the measurement and evaluation methods reported nowadays are not standardized so that it is difficult to compare results reported by different people/methods.^15^ Particularly, the electrochemical performances reported in most of the studies are only normalized to the superficial geometric electrode area, but the specific surface morphology, effective surface area, as well as the catalyst loading quantity are either ignored or not aligned to make effective comparison. In addition, it is known that the nanocrystals with high‐indexed facets can have much improved catalytic activity, while the realization of these high‐indexed facets in low‐dimensional (e.g. 1D and 2D) structures is still substantially challenging. Thus, the design and optimization of those new catalysts should be in a large span of length scales, from atomic, nanoscale, to micro‐ and even macroscopic levels. The aims of these designs are to achieve the minimum free energy change for the reaction pathway on a given surface, while at the same time to optimize the effective surface area/sites for electrochemical reactions. In addition, the measurement methods should also be standardized and evaluated by third‐parties to get valuable comparison across different results.

Last but not least, the capabilities of interfacing these electrocatalysts with semiconductor or molecular photoabsorbers, as well as large‐scale synthesis and implementation of these electrocatalytic or photo‐electrocatalytic materials, have yet to be demonstrated. The ultimate efficiency of the photoconversion system is determined by the nanoscale interfacial properties between semiconductor photoabsorbers and electrocatalysts, and thus the understanding and perfect control of these interfaces with exquisite tuning of charge separation and transfer are critical. In addition, the scale‐up of synthesis and utilization of these catalyst materials with controlled size, morphology, composition and electrochemical properties need to be established. It should be aware that even though earth‐abundant materials are used as low‐cost precursors, the synthetic routes of these materials may not be cost effective or hard to be implemented in large scales. The total cost of the whole synthesis and electrochemical process, including the control of waste and acid/base pollution, should be carefully put into consideration and well optimized, in order to realize efficient and robust deployment of large‐scale water splitting for sustainable energy requirement globally.

## References

[1] M. G. Walter , E. L. Warren , J. R. McKone , S. W. Boettcher , Q. X. Mi , E. A. Santori , N. S. Lewis , Chem. Rev. 2010, 110, 6446. 21062097 10.1021/cr1002326

[2] C. Liu , B. C. Colon , M. Ziesack , P. A. Silver , D. G. Nocera , Science 2016, 352, 1210. 27257255 10.1126/science.aaf5039

[3] J. Luo , J. H. Im , M. T. Mayer , M. Schreier , M. K. Nazeeruddin , N. G. Park , S. D. Tilley , H. J. Fan , M. Gratzel , Science 2014, 345, 1593. 25258076 10.1126/science.1258307

[4] K. K. Sakimoto , A. B. Wong , P. D. Yang , Science 2016, 351, 74. 26721997 10.1126/science.aad3317

[5] X. B. Chen , S. H. Shen , L. J. Guo , S. S. Mao , Chem. Rev. 2010, 110, 6503. 21062099 10.1021/cr1001645

[6] C. G. Morales‐Guio , L. A. Stern , X. Hu , Chem. Soc. Rev. 2014, 43, 6555. 24626338 10.1039/c3cs60468c

[7] J. O. M. Bockris , Int. J. Hydrogen Energy 2013, 38, 2579.

[8] Y. Jiao , Y. Zheng , M. Jaroniec , S. Z. Qiao , Chem. Soc. Rev. 2015, 44, 2060. 25672249 10.1039/c4cs00470a

[9] W. Kim , B. A. McClure , E. Edri , H. Frei , Chem. Soc. Rev. 2016, 45, 3221. 27121982 10.1039/c6cs00062b

[10] J. Ran , J. Zhang , J. Yu , M. Jaroniec , S. Z. Qiao , Chem. Soc. Rev. 2014, 43, 7787. 24429542 10.1039/c3cs60425j

[11] Y. Shi , B. Zhang , Chem. Soc. Rev. 2016, 45, 1529. 26806563 10.1039/c5cs00434a

[12] Y. Xu , M. Kraft , R. Xu , Chem. Soc. Rev. 2016, 45, 3039. 27094875 10.1039/c5cs00729a

[13] Y. Xu , B. Zhang , Chem. Soc. Rev. 2014, 43, 2439. 24458336 10.1039/c3cs60351b

[14] C. Ye , M. D. Regulacio , S. H. Lim , S. Li , Q. H. Xu , M. Y. Han , Chem. Eur. J. 2015, 21, 9514. 25982850 10.1002/chem.201500781

[15] X. Zou , Y. Zhang , Chem. Soc. Rev. 2015, 44, 5148. 25886650 10.1039/c4cs00448e

[16] A. J. F. Bard , R. Larry , Electrochemical Methods: Fundamentals and Applications. John Wiley & Sons, Inc, New York, USA 2001

[17] M. S. Faber , S. Jin , Energy Environ. Sci. 2014, 7, 3519.

[18] W. T. Hong , M. Risch , K. A. Stoerzinger , A. Grimaud , J. Suntivich , Y. Shao‐Horn , Energy Environ. Sci. 2015, 8, 1404.

[19] G. Wu , P. Zelenay , Acc. Chem. Res. 2013, 46, 1878. 23815084 10.1021/ar400011z

[20] S. J. Guo , S. Zhang , S. H. Sun , Angew. Chem. Int. Ed. 2013, 52, 8526. 10.1002/anie.201207186 23775769

[21] M. Armand , J. M. Tarascon , Nature 2008, 451, 652. 18256660 10.1038/451652a

[22] L. Johnson , C. M. Li , Z. Liu , Y. H. Chen , S. A. Freunberger , P. C. Ashok , B. B. Praveen , K. Dholakia , J. M. Tarascon , P. G. Bruce , Nat. Chem. 2014, 6, 1091. 25411888 10.1038/nchem.2101

[23] J. Li , Y. C. Wang , T. Zhou , H. Zhang , X. H. Sun , J. Tang , L. J. Zhang , A. M. Al‐Enizi , Z. Q. Yang , G. F. Zheng , J. Am. Chem. Soc. 2015, 137, 14305. 26496655 10.1021/jacs.5b07756

[24] J. Greeley , T. F. Jaramillo , J. Bonde , I. B. Chorkendorff , J. K. Norskov , Nat. Mater. 2006, 5, 909. 17041585 10.1038/nmat1752

[25] J. Wang , W. Cui , Q. Liu , Z. Xing , A. M. Asiri , X. Sun , Adv. Mater. 2016, 28, 215. 26551487 10.1002/adma.201502696

[26] Y. Li , P. Hasin , Y. Wu , Adv. Mater. 2010, 22, 1926. 20526996 10.1002/adma.200903896

[27] M. S. Faber , R. Dziedzic , M. A. Lukowski , N. S. Kaiser , Q. Ding , S. Jin , J. Am. Chem. Soc. 2014, 136, 10053. 24901378 10.1021/ja504099w

[28] Y. L. Wang , T. Y. Wang , P. M. Da , M. Xu , H. Wu , G. F. Zheng , Adv. Mater. 2013, 25, 5177. 23828226 10.1002/adma.201301943

[29] Y. D. Su , C. Liu , S. Brittman , J. Y. Tang , A. Fu , N. Kornienko , Q. Kong , P. D. Yang , Nat. Nanotechnol. 2016, 11, 609. 27018660 10.1038/nnano.2016.30

[30] B. Tian , T. J. Kempa , C. M. Lieber , Chem. Soc. Rev. 2009, 38, 16. 19088961 10.1039/b718703n

[31] T. J. Kempa , R. W. Day , S. K. Kim , H. G. Park , C. M. Lieber , Energy Environ. Sci. 2013, 6, 719.

[32] H. Wu , Y. Cui , Nano Today 2012, 7, 414.

[33] C. K. Chan , H. L. Peng , G. Liu , K. McIlwrath , X. F. Zhang , R. A. Huggins , Y. Cui , Nat. Nanotechnol. 2008, 3, 31. 18654447 10.1038/nnano.2007.411

[34] A. I. Hochbaum , P. D. Yang , Chem. Rev. 2010, 110, 527. 19817361 10.1021/cr900075v

[35] Z. Peng , D. S. Jia , A. M. Al‐Enizi , A. A. Elzatahry , G. F. Zheng , Adv. Energy Mater. 2015, 5, 1402031.

[36] X. W. Liu , W. W. Li , H. Q. Yu , Chem. Soc. Rev. 2014, 43, 7718. 23959403 10.1039/c3cs60130g

[37] J. Tian , Q. Liu , A. M. Asiri , X. Sun , J. Am. Chem. Soc. 2014, 136, 7587. 24830333 10.1021/ja503372r

[38] P. Jiang , Q. Liu , Y. Liang , J. Tian , A. M. Asiri , X. Sun , Angew. Chem. Int. Ed. 2014, 53, 12855. 10.1002/anie.201406848 25257101

[39] J. Tian , Q. Liu , N. Cheng , A. M. Asiri , X. Sun , Angew. Chem. Int. Ed. 2014, 53, 9577. 10.1002/anie.201403842 25044801

[40] J. Tian , N. Cheng , Q. Liu , W. Xing , X. Sun , Angew. Chem. Int. Ed. 2015, 54, 5493. 10.1002/anie.201501237 25721096

[41] Q. Ding , F. Meng , C. R. English , M. Caban‐Acevedo , M. J. Shearer , D. Liang , A. S. Daniel , R. J. Hamers , S. Jin , J. Am. Chem. Soc. 2014, 136, 8504. 24892384 10.1021/ja5025673

[42] M. A. Lukowski , A. S. Daniel , F. Meng , A. Forticaux , L. Li , S. Jin , J. Am. Chem. Soc. 2013, 135, 10274. 23790049 10.1021/ja404523s

[43] J. Kibsgaard , Z. Chen , B. N. Reinecke , T. F. Jaramillo , Nat. Mater. 2012, 11, 963. 23042413 10.1038/nmat3439

[44] J. D. Benck , Z. Chen , L. Y. Kuritzky , A. J. Forman , T. F. Jaramillo , ACS Catalysis 2012, 2, 1916.

[45] D. Voiry , H. Yamaguchi , J. Li , R. Silva , D. C. B. Alves , T. Fujita , M. Chen , T. Asefa , V. B. Shenoy , G. Eda , M. Chhowalla , Nat. Mater. 2013, 12, 850. 23832127 10.1038/nmat3700

[46] M. A. Lukowski , A. S. Daniel , C. R. English , F. Meng , A. Forticaux , R. J. Hamers , S. Jin , Energy Environ. Sci. 2014, 7, 2608.

[47] E. J. Popczun , J. R. McKone , C. G. Read , A. J. Biacchi , A. M. Wiltrout , N. S. Lewis , R. E. Schaak , J. Am. Chem. Soc. 2013, 135, 9267. 23763295 10.1021/ja403440e

[48] E. J. Popczun , C. G. Read , C. W. Roske , N. S. Lewis , R. E. Schaak , Angew. Chem. Int. Ed. 2014, 53, 5427. 10.1002/anie.201402646 24729482

[49] J. R. McKone , B. F. Sadtler , C. A. Werlang , N. S. Lewis , H. B. Gray , ACS Catalysis 2013, 3, 166.

[50] J. R. McKone , E. L. Warren , M. J. Bierman , S. W. Boettcher , B. S. Brunschwig , N. S. Lewis , H. B. Gray , Energy Environ. Sci. 2011, 4, 3573.

[51] J. M. Wang , Y. M. Zheng , F. Q. Nie , J. Zhai , L. Jiang , Langmuir 2009, 25, 14129. 19583224 10.1021/la9010828

[52] M. Caban‐Acevedo , M. L. Stone , J. R. Schmidt , J. G. Thomas , Q. Ding , H. C. Chang , M. L. Tsai , J. H. He , S. Jin , Nat. Mater. 2015, 14, 1245. 26366849 10.1038/nmat4410

[53] W. F. Chen , J. T. Muckerman , E. Fujita , Chem. Comm. 2013, 49, 8896. 23982806 10.1039/c3cc44076a

[54] L. Liao , S. N. Wang , J. J. Xiao , X. J. Bian , Y. H. Zhang , M. D. Scanlon , X. L. Hu , Y. Tang , B. H. Liu , H. H. Girault , Energy Environ. Sci. 2014, 7, 387.

[55] S. Chen , J. Duan , M. Jaroniec , S. Z. Qiao , Adv. Mater. 2014, 26, 2925. 24510748 10.1002/adma.201305608

[56] M. Gong , W. Zhou , M. C. Tsai , J. Zhou , M. Guan , M. C. Lin , B. Zhang , Y. Hu , D. Y. Wang , J. Yang , S. J. Pennycook , B. J. Hwang , H. Dai , Nat. Commun. 2014, 5, 4695. 25146255 10.1038/ncomms5695

[57] X. Zou , X. Huang , A. Goswami , R. Silva , B. R. Sathe , E. Mikmekova , T. Asefa , Angew. Chem. Int. Ed. 2014, 126, 4461. 10.1002/anie.201311111 24652809

[58] D. J. Li , U. N. Maiti , J. Lim , D. S. Choi , W. J. Lee , Y. Oh , G. Y. Lee , S. O. Kim , Nano Lett. 2014, 14, 1228. 24502837 10.1021/nl404108a

[59] J. Deng , P. J. Ren , D. H. Deng , L. Yu , F. Yang , X. H. Bao , Energy Environ. Sci. 2014, 7, 1919.

[60] Z. Xing , Q. Liu , W. Xing , A. M. Asiri , X. Sun , ChemSusChem 2015, 8, 1850. 25916622 10.1002/cssc.201500138

[61] Q. Liu , J. Tian , W. Cui , P. Jiang , N. Cheng , A. M. Asiri , X. Sun , Angew. Chem. Int. Ed. 2014, 126, 6828. 10.1002/anie.201404161 24845625

[62] D. Y. Wang , M. Gong , H. L. Chou , C. J. Pan , H. A. Chen , Y. Wu , M. C. Lin , M. Guan , J. Yang , C. W. Chen , Y. L. Wang , B. J. Hwang , C. C. Chen , H. Dai , J. Am. Chem. Soc. 2015, 137, 1587. 25588180 10.1021/ja511572q

[63] W. Cui , Q. Liu , N. Cheng , A. M. Asiri , X. Sun , Chem. Comm. 2014, 50, 9340. 25000967 10.1039/c4cc02713b

[64] M. Shalom , S. Gimenez , F. Schipper , I. Herraiz‐Cardona , J. Bisquert , M. Antonietti , Angew. Chem. Int. Ed. 2014, 126, 3728. 10.1002/anie.201309415 24574144

[65] Y. Zheng , Y. Jiao , Y. Zhu , L. H. Li , Y. Han , Y. Chen , A. Du , M. Jaroniec , S. Z. Qiao , Nat. Commun. 2014, 5, 3783. 24769657 10.1038/ncomms4783

[66] N. Holmberg , K. Laasonen , J. Phys. Chem. C 2015, 119, 16166. 10.1021/acs.jpclett.5b01846 26722898

[67] N. Holmberg , K. Laasonen , J. Phys. Chem. Lett. 2015, 6, 3956. 26722898 10.1021/acs.jpclett.5b01846

[68] Y. Matsumoto , E. Sato , Mater. Chem. Phys. 1986, 14, 397.

[69] Y. Gorlin , T. F. Jaramillo , J. Am. Chem. Soc. 2010, 132, 13612. 20839797 10.1021/ja104587v

[70] W. J. Zhou , X. J. Wu , X. H. Cao , X. Huang , C. L. Tan , J. Tian , H. Liu , J. Y. Wang , H. Zhang , Energy Environ. Sci. 2013, 6, 2921.

[71] I. Nikolov , R. Darkaoui , E. Zhecheva , R. Stoyanova , N. Dimitrov , T. Vitanov , J. Electroanal. Chem. 1997, 429, 157.

[72] X. J. Liu , Z. Chang , L. Luo , T. H. Xu , X. D. Lei , J. F. Liu , X. M. Sun , Chem. Mater. 2014, 26, 1889.

[73] Y. Wang , T. Zhou , K. Jiang , P. Da , Z. Peng , J. Tang , B. Kong , W.‐B. Cai , Z. Yang , G. Zheng , Adv. Energy Mater. 2014, 4, 1400696.

[74] X. B. Chen , L. Liu , P. Y. Yu , S. S. Mao , Science 2011, 331, 746. 21252313 10.1126/science.1200448

[75] G. M. Wang , H. Y. Wang , Y. C. Ling , Y. C. Tang , X. Y. Yang , R. C. Fitzmorris , C. C. Wang , J. Z. Zhang , Y. Li , Nano Lett. 2011, 11, 3026. 21710974 10.1021/nl201766h

[76] G. M. Wang , Y. C. Ling , H. Y. Wang , X. Y. Yang , C. C. Wang , J. Z. Zhang , Y. Li , Energy Environ. Sci. 2012, 5, 6180.

[77] Y. Meng , W. Song , H. Huang , Z. Ren , S. Y. Chen , S. L. Suib , J. Am. Chem. Soc. 2014, 136, 11452. 25058174 10.1021/ja505186m

[78] Y. C. Wang , K. Jiang , H. Zhang , T. Zhou , J. W. Wang , W. Wei , Z. Q. Yang , X. H. Sun , W. B. Cai , G. F. Zheng , Adv. Sci. 2015, 2, 1500003. 10.1002/advs.201500003 PMC5024083 27668150

[79] W. Wei , Y. C. Wang , H. Wu , A. M. Al‐Enizi , L. J. Zhang , G. F. Zheng , Nanotechnol. 2016, 27, LT201. 10.1088/0957-4484/27/2/02LT01 26629880

[80] S. W. Li , Y. C. Wang , S. J. Peng , L. J. Zhang , A. M. Al‐Enizi , H. Zhang , X. H. Sun , G. F. Zheng , Adv. Energy Mater. 2016, 6, 1501661.

[81] Z. Chen , A. R. Rathmell , S. Ye , A. R. Wilson , B. J. Wiley , Angew. Chem. Int. Ed. 2013, 52, 13708. 10.1002/anie.201306585 24136831

[82] Z. L. Zhao , H. X. Wu , H. L. He , X. L. Xu , Y. D. Jin , Adv. Funct. Mater. 2014, 24, 4698.

[83] A. Zhao , J. Masa , W. Xia , A. Maljusch , M. G. Willinger , G. Clavel , K. Xie , R. Schlogl , W. Schuhmann , M. Muhler , J. Am. Chem. Soc. 2014, 136, 7551. 24815686 10.1021/ja502532y

[84] J. Geng , H. Wu , A. M. Al‐Enizi , A. A. Elzatahry , G. Zheng , Nanoscale 2015, 7, 14378. 26247312 10.1039/c5nr04603c

[85] G. L. Tian , Q. Zhang , B. S. Zhang , Y. G. Jin , J. Q. Huang , D. S. Su , F. Wei , Adv. Funct. Mater. 2014, 24, 5956.

[86] X. Lu , W. L. Yim , B. H. Suryanto , C. Zhao , J. Am. Chem. Soc. 2015, 137, 2901. 25658670 10.1021/ja509879r

[87] Z. Peng , S. W. Yang , D. S. Jia , P. M. Da , P. He , A. M. Al‐Enizi , G. Q. Ding , X. M. Xie , G. F. Zheng , J. Mater. Chem. A 2016, 4, 12878.

[88] S. Cobo , J. Heidkamp , P. A. Jacques , J. Fize , V. Fourmond , L. Guetaz , B. Jousselme , V. Ivanova , H. Dau , S. Palacin , M. Fontecave , V. Artero , Nat. Mater. 2012, 11, 802. 22863815 10.1038/nmat3385

[89] X. Yu , T. Hua , X. Liu , Z. Yan , P. Xu , P. Du , ACS Appl Mater Interfaces 2014, 6, 15395. 25136924 10.1021/am503938c

[90] J. Wang , D. F. Gao , G. X. Wang , S. Miao , H. H. Wu , J. Y. Li , X. H. Bao , J. Mater. Chem. A 2014, 2, 20067.

[91] Y. N. Regmi , C. Wan , K. D. Duffee , B. M. Leonard , ChemCatChem 2015, 7, 3911.

[92] J. Q. Tian , N. Y. Cheng , Q. Liu , X. P. Sun , Y. Q. He , A. M. Asiri , J. Mater. Chem. A 2015, 3, 20056.

[93] P. Chen , K. Xu , S. Tao , T. Zhou , Y. Tong , H. Ding , L. Zhang , W. Chu , C. Wu , Y. Xie , Adv. Mater. 2016, 28, 7527. 27309389 10.1002/adma.201601663

[94] C. Tang , N. Cheng , Z. Pu , W. Xing , X. Sun , Angew. Chem. Int. Ed. 2015, 54, 9351. 10.1002/anie.201503407 26136347

[95] D. Liu , Q. Lu , Y. Luo , X. Sun , A. M. Asiri , Nanoscale 2015, 7, 15122. 26355688 10.1039/c5nr04064g

[96] A. Sivanantham , P. Ganesan , S. Shanmugam , Adv. Funct. Mater. 2016, 26, 4661.

[97] W. Z. Fang , D. N. Liu , Q. Lu , X. P. Sun , A. M. Asiri , Electrochem. Commun. 2016, 63, 60.

[98] R. Xu , R. Wu , Y. M. Shi , J. F. Zhang , B. Zhang , Nano Energy 2016, 24, 103.

[99] Y. P. Zhu , Y. P. Liu , T. Z. Ren , Z. Y. Yuan , Adv. Funct. Mater. 2015, 25, 7337.

[100] Q. T. Zhang , Y. H. Wang , Y. C. Wang , A. M. Al‐Enizi , A. A. Elzatahry , G. F. Zheng , J. Mater. Chem. A 2016, 4, 5713.

[101] M. Tahir , N. Mahmood , X. X. Zhang , T. Mahmood , F. K. Butt , I. Aslam , M. Tanveer , F. Idrees , S. Khalid , I. Shakir , Y. M. Yan , J. J. Zou , C. B. Cao , Y. L. Hou , Nano Res. 2015, 8, 3725.

[102] X. H. Gao , H. X. Zhang , Q. G. Li , X. G. Yu , Z. L. Hong , X. W. Zhang , C. D. Liang , Z. Lin , Angew. Chem. Int. Ed. 2016, 128, 6398.

